# Streptokinase is dispensable in *Streptococcus dysgalactiae* subspecies *equisimilis* infections of human dendritic cells

**DOI:** 10.1038/s41598-025-87404-x

**Published:** 2025-01-21

**Authors:** Katharina E. Folz, Nikolai Siemens

**Affiliations:** Department of Molecular Genetics and Infection Biology, University of Greifswald, 17489 Greifswald, Germany

**Keywords:** *Streptococcus dysgalactiae* subspecies *equisimilis*, Streptokinase, Dendritic cells, Pathogens, Infection

## Abstract

In recent years, increased numbers of severe *Streptococcus dysgalactiae* subsp. *equisimilis* (SDSE) infections, including necrotizing soft tissue infections (NSTIs), have been reported. One of the main virulence factors of SDSE is streptokinase (Ska). Ska promotes bacterial spread in the tissue through Ska-plasminogen interactions and subsequent activation of plasminogen to plasmin. In this study, the impact of streptokinase on SDSE infections of human monocyte-derived dendritic cells (moDCs) was investigated. MoDCs were infected with SDSE strain S118 and its isogenic mutant lacking streptokinase. All infections were performed with and without human serum to compare direct Ska-mediated as well as plasmin activity-related effects. Intracellular killing kinetics, moDC viability and maturation, as well as the release of pro-inflammatory cytokines were assessed. Irrespective of the strain and experimental conditions, the bacteria were equally phagocytosed and killed. MoDCs remained viable, readily matured and secreted equal amounts of cytokines in response to S118 as well as S118Δ*ska* infections. Our data demonstrate that moDCs response to SDSE infections is not affected by Ska or its respective plasminogen activating function.

**Supplementary Information:**

The online version contains supplementary material available at 10.1038/s41598-025-87404-x.

## Introduction

*Streptococcus dysgalactiae* subspecies *equisimilis* (SDSE) is an opportunistic commensal of the human skin, upper respiratory tract, gastrointestinal tract, and genital tract^1^. Although considered less pathogenic than other β-hemolytic streptococci, recent studies have shown that SDSE is an emerging cause of bacteremia and necrotizing soft tissue infections (NSTIs)^2–6^ with relatively high mortality rates^1,2,7^. SDSE is genetically closely related to *Streptococcus pyogenes*, resulting in the expression of analogous virulence factors, which include the M protein, streptolysin O, streptolysin S, and streptokinase (Ska), among others^1,8,9^.

The function of Ska in *S. pyogenes* and SDSE is well characterized. It binds exclusively to human plasminogen, leading to the formation of an equimolar Ska-plasminogen complex and its activation^10,11^. The activated complex converts further plasminogen into the enzymatically active form plasmin^11,12^. Plasmin, in turn, proteolytically cleaves fibrin and degrades extracellular matrix proteins^13^. By exploiting such host functions, the bacteria escape the innate immune response and spread through the tissue^14^. Furthermore, recent studies have shown that (i) invasive SDSE strains are characterized by a higher Ska activity as compared to non-invasive strains^15^, (ii) Ska has an impact on metabolic activity of SDSE^16^, and (iii) Ska itself prevents SDSE from entering the biofilm stage^16^.

Through their ability to recognize, ingest, and process pathogens and present antigens, dendritic cells (DC) connect the innate and adaptive arms of the immune system^17–20^. Such processes lead to the maturation of DCs^17,18^. Subsequently, DCs migrate to the lymph nodes and present antigens to T cells via major histocompatibility complex (MHC) II molecules. In addition, various co-stimulatory molecules are increasingly expressed in mature DCs^17–20^. So far, there is no data on DC-SDSE interactions. In *S. pyogenes* infections, it was shown that capsule and streptolysin O are potent inhibitors of DC maturation^21^. Furthermore, DCs limit the spread of *S. pyogenes* in murine model infections^22^.

In this study, we investigated the impact of Ska on SDSE-DC interactions using primary human monocyte-derived DCs (moDCs). We show that Ska plays no role in moDC infections. MoDCs easily eradicated intracellular bacteria, remained viable, readily matured, and secreted cytokines in response to all SDSE infections.

## Results

### MoDCs kill intracellular SDSE, remain viable, and readily mature in response to infections

**Fig. 1:**
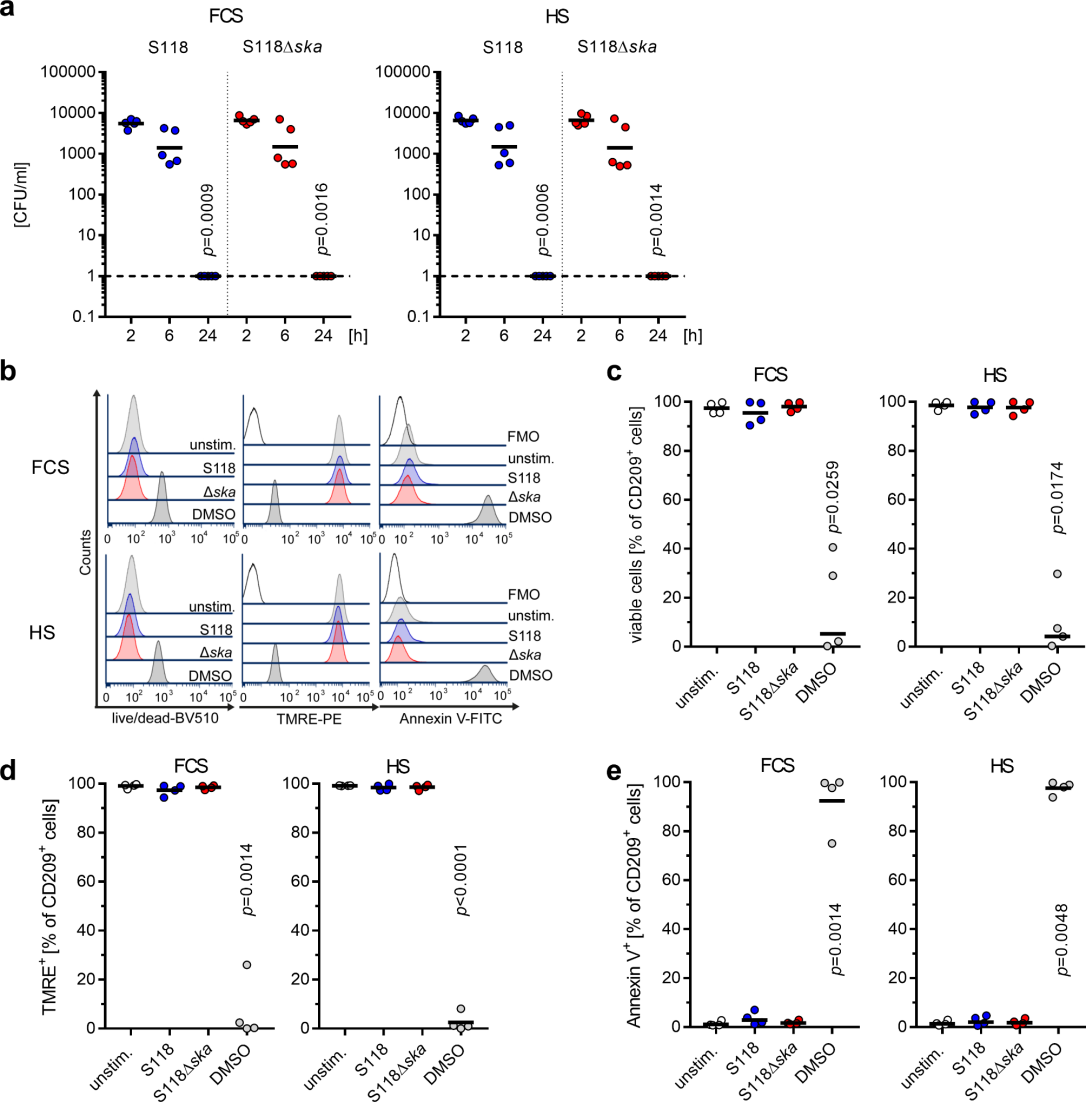
MoDCs kill intracellular S118 and S118∆*ska* and remain viable. MoDCs were infected with S118 or S118Δ*ska* (MOI10). After 1 h of infection, antibiotics were added to kill extracellular bacteria. (**a**) To assess intracellular killing of S118 and S118∆*ska*, the CFU was determined 2 h, 6 h, and 24 h post infection. Furthermore, moDC viability was assessed after 24 h (**b-e**, *n* = 4). (**b**) Representative histograms and analyses of viable moDCs (**c**) with intact mitochondrial membrane potential (**d**), and extracellular phosphatidyl serine (**e**). All infections were performed in presence of FCS or human serum (HS). Each dot represents one independent experiment with cells from one donor (*n* ≥ 4). Horizontal lines denote geometric mean values. Dashed lines in (a) represent 0 values. The level of significance was determined using Kruskal-Wallis test.

To assess the impact of Ska on moDCs, the uptake of bacteria, intracellular killing kinetics, moDC viability, maturation and secretion of cytokines were determined in response to infection. To analyze the direct impact of Ska and/or its plasminogen converting activity, all experiments were carried out in presence of fetal calf serum (FCS) or human serum, respectively. MoDCs were infected with S118 and S118∆*ska* for 1 h followed by antibiotic treatment for up to 23 h. Intracellular bacterial amounts were determined 2 h, 6 h, and 24 h post infections. In all experimental conditions, no differences in bacterial uptake as well as in killing kinetics were observed (Fig. 1a). Furthermore, the viability of moDCs was assessed 24 h post infection (Fig. 1b-e). Regardless of the experimental conditions as well as the strain used, infected moDCs showed (i) high frequencies of viable cells (Fig. 1c), (ii) high frequencies of cells with intact mitochondrial membrane potential (TMRE; Fig. 1d), and (iii) negligible levels of extracellular phosphatidyl serine (Annexin V; Fig. 1e).

**Fig. 2:**
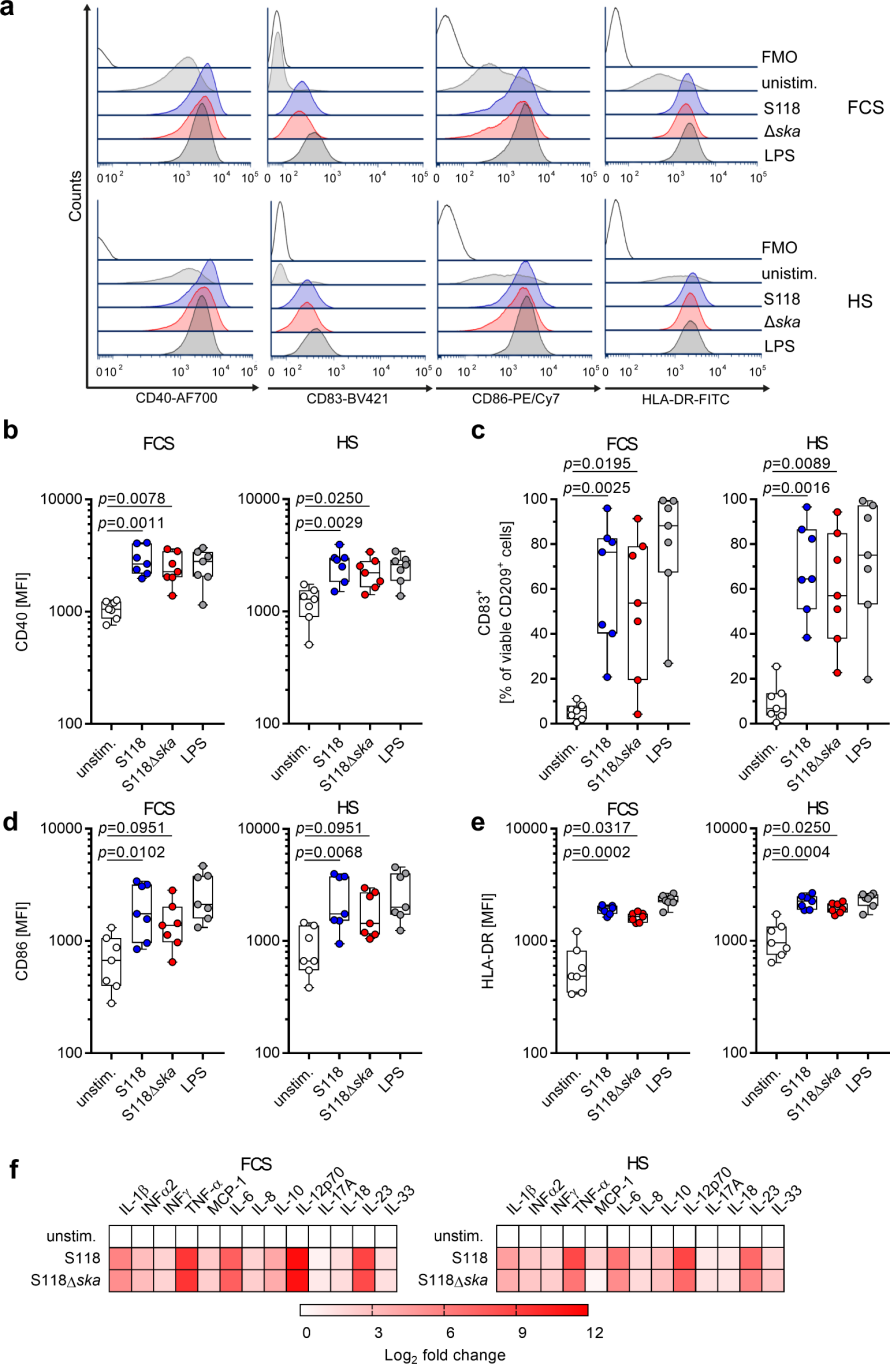
MoDCs mature and secrete cytokines in response to SDSE infections. MoDCs were infected with SDSE S118 and S118Δ*ska* (MOI10). After 1 h, extracellular bacteria were killed. After a total of 24 h of infections, cells were stained and analyzed via flow cytometry to assess the influence of Ska on moDC maturation (**a-e**). Representative histograms for each marker are shown in (**a**). The maturation was evaluated by assessing the expression of surface markers CD40 (**b**), CD83 (**c**), CD86 (**d**) and HLA-DR (**e**). Moreover, released cytokines were measured after 24 h of infection using a multiplex assay (**f**).The heat maps display log2 fold change in cytokine concentration compared to unstimulated controls. Original cytokine data are presented in Table S1. All infections were performed in presence of FCS or human serum (HS). The data in (**b-e**) are presented as boxplots. Each dot represents one independent experiment with cells from one donor (*n* = 7). The level of significance was determined using Kruskal-Wallis test followed by Dunn’s multiple comparison test.

Next, Ska impact on moDC maturation and cytokine secretion was assessed (Fig. 2a-e). Therefore, moDCs were infected with S118 and S118Δ*ska* as described above and were analyzed 24 h post infection (Fig. 2). In all infectious conditions, moDCs readily matured characterized by increased frequencies of CD83^+^ cells as well as upregulation of CD40, CD86, and HLA-DR (Fig. 2a-e). Finally, the release of cytokines/chemokines in response to infections was determined. Regardless of the strain used and infectious condition (FCS vs. human serum) infected moDCs equally responded by releasing mostly comparable amounts of all 13 cytokines/chemokines analyzed (Fig. 2f, Table S1). However, a trend of reduced secretion of IL1β, TNF-α, IL-12p70, and IL-23 in response to S118Δ*ska* infections in the presence of human serum was observed, although not statistically significant.

## Discussion

In recent years, SDSE has emerged as a rising cause of severe clinical manifestations such as bacteremia and NSTIs^2–6^. An important virulence factor for SDSE is Ska. It allows the pathogen to spread and invade deeper layers through activation of plasminogen^10,11^. Furthermore, it was demonstrated that invasive SDSE strains are characterized by increased Ska activity as compared to non-invasive strains^15^ and that Ska has a negative impact on SDSE biofilm formation^16^. Here, we show that Ska itself as well as its function play no role during interaction of SDSE with moDC. MoDCs eradicated intracellular SDSE and readily matured in response to all infections irrespective of the chosen condition.

DCs are important immune sentinels involved in recognition and clearance of pathogens, recruitment of other immune cells, and antigen presentation^17–20^. In order to accomplish their function during an infection, it is important that DCs remain viable, mature, secrete cytokines and thus, orchestrate the adaptive immune response^17–20^. Our analyses revealed that Ska is not interfering with these processes. In fact, the bacteria were equally phagocytosed and killed, while the moDCs remained viable in all infectious conditions. This indicates that neither the deletion of Ska nor its activity has an impact on such processes. In contrast, it was shown for keratinocytes that the presence of Ska is associated with a decreased cell invasion^23^. In contrast to keratinocytes, which are actively invaded by bacteria, phagocytes are actively ingesting pathogens. Our data suggest that in contrast to bacteria-mediated invasion of epithelial cells, Ska plays no role in phagocytosis^17^. The viability of moDCs was likewise unaffected in all infectious conditions, confirming their robustness in the defence of streptococcal infections^24,25^.

Furthermore, maturation and cytokine secretion are crucial for modulating the adaptive immune response^17,18^. Our analyses revealed that moDCs equally upregulate the expression of MHCII and the costimulatory molecules on their surface and secrete equal amounts of cytokines/chemokines in response to SDSE infections. In addition, no differences between the experimental conditions were detected. This is in line with previously published data, which show that moDCs rather equally respond to streptococcal infections and if differences are found, they are most likely induced by regulatory elements and not secreted factors like Ska^24^.

In conclusion, Ska does not impact moDC response to SDSE infections. However, to the best of our knowledge, this is the first report focusing on SDSE and moDCs interactions. Future studies should aim to identify potential new roles of Ska (e.g., in SDSE biofilms^16^) in SDSE infections.

## Methods

### Ethics declarations

Buffy coats of blood provided by the blood bank at the University Medicine Greifswald were used. The buffy coats were provided anonymously. The ethical research committee at the University Medicine Greifswald approved the study (Ref. No. BB 014/14). All experiments were carried out in accordance with the approved guidelines.

### Bacterial strains

SDSE strain S118 (invasive soft tissue infection isolate; *emm*-type: stG10.0; FCT-type: 6a; ST15) collected from a patient identified by Haukeland University Hospital, Bergen (Norway) between 2003 and 2013 was used^3,4,6^. Construction of S118Δ*ska* was previously published^16^. Both strains were cultured in Todd Hewitt Broth (THY, Roth) supplemented with 1.5% (w/v) yeast extract (Roth) at 37 °C and 5% CO_2_.

### Isolation of human monocytes and generation of dendritic cells

Human monocytes were isolated from buffy coats using the EasySep Human CD14 Positive Selection Kit II (Stemcell™ Technologies) according to manufacturer’s instructions. Monocytes were incubated for 5 d in RPMI1640 (Cytivia) medium supplemented with 10% (v/v) heat-inactivated fetal calf serum (Thermo Fischer), 89 ng/ml GM-CSF (Immunotools), and 22 ng/ml IL-4 (Immunotools) to generate moDCs. After 3 d, the medium was exchanged.

### Infections of dendritic cells

All infections were performed in RPMI1640 complete medium containing either 10% FCS or human serum (AB male donor; Merck). 1 × 10^5^ moDCs were infected with S118 or S118∆*ska* from over-night cultures (16–18 h) at a multiplicity of infection (MOI) 10. Extracellular bacteria were killed 1 h after infection by adding RPMI1640 supplemented with 100 µg/ml streptomycin and 100 IU/ml penicillin G (Hyclone). After 24 h post infection, supernatants were collected and stored at -80 °C until further analysis. MoDCs were directly prepared for flow cytometry.

For assessing intracellular bacterial survival kinetics, 1 × 10^5^ moDCs were infected as described above. After addition of antibiotics, cells were washed, lysed, and intracellular bacteria were plated on blood agar plates (Oxoid) 2 h, 6 h, and 24 h post infection.

### Flow cytometry

Dead cells were labeled using the Zombie Aqua Fixable Viability Kit (BioLegend). Unspecific binding of immunoglobulins was blocked by using Human TruStain FcX (BioLegend) according to the manufacturer’s instructions. Incubations of cells with titrated amounts of monoclonal antibodies as the well as TMRE-Mitochondrial Membrane Potential Assay Kit (Abcam) and FITC Annexin V (BioLegend) were carried out for 30 min at 4 °C in the dark. Cells were washed between each staining step and fixed using the Cyto-Fast Fix/Perm Buffer Set (Bio-Legend). Antibodies and clones directed against the following markers were used (target, clone, fluorochrome, all Biolegend): CD209 (9E9A8, APC), CD209 (9E9A8, BV421), CD40 (5C3, AlexaFluor 700), CD83 (HB15e, BV421), CD86 (BU63, PE/Cyanine7), and MHCII (L243, FITC). Data were acquired using a FACSAria III flow cytometer and FACS DIVA 8.0 Software (both BD Biosciences) and analyzed using FCS Express 7 Software (De Novo Software). The gating strategy to identify moDC is presented in the supplemental information (Figure S1).

### Cytokine measurements

Cytokine concentrations in cell culture supernatants were measured via LEGENDPlex human inflammation panel 1 (13-plex) kit (BioLegend) according to the manufacturer’s instructions. Data were acquired with a FACSAria III flow cytometer using FACS DIVA Software (both BD Bioscience) and analyzed using LEGENDPlex software (BioLegend).

### Statistics

Statistical significance of differences was determined using Kruskal-Wallis test with Dunn’s multiple comparison posttest. Statistics were performed using GraphPad Prism version 7. A *p* value less than 0.05 was considered significant.

## Electronic supplementary material

Below is the link to the electronic supplementary material.

Supplementary Material 1

## Data Availability

All Data related to this study are included in the manuscript and is available from the corresponding author upon reasonable request.
