# Supplementary material for: Streptokinase is dispensable in Streptococcus dysgalactiae subspecies equisimilis infections of human dendritic cells
Source: Sci Rep. 2025 Jan 21;15:2723. doi: 10.1038/s41598-025-87404-x (PMC11751451; doi:10.1038/s41598-025-87404-x)
Supplement: Supplementary file 1 — Supplementary Material 1 [file 41598_2025_87404_MOESM1_ESM.pdf]

Supplementary Information

Streptokinase is dispensable in *Streptococcus dysgalactiae* subspecies *equisimilis* infections of human dendritic cells

Katharina E. Folz[1] and Nikolai Siemens[1]*

[1]Department of Molecular Genetics and Infection Biology, University of Greifswald, 17489 Greifswald, Germany

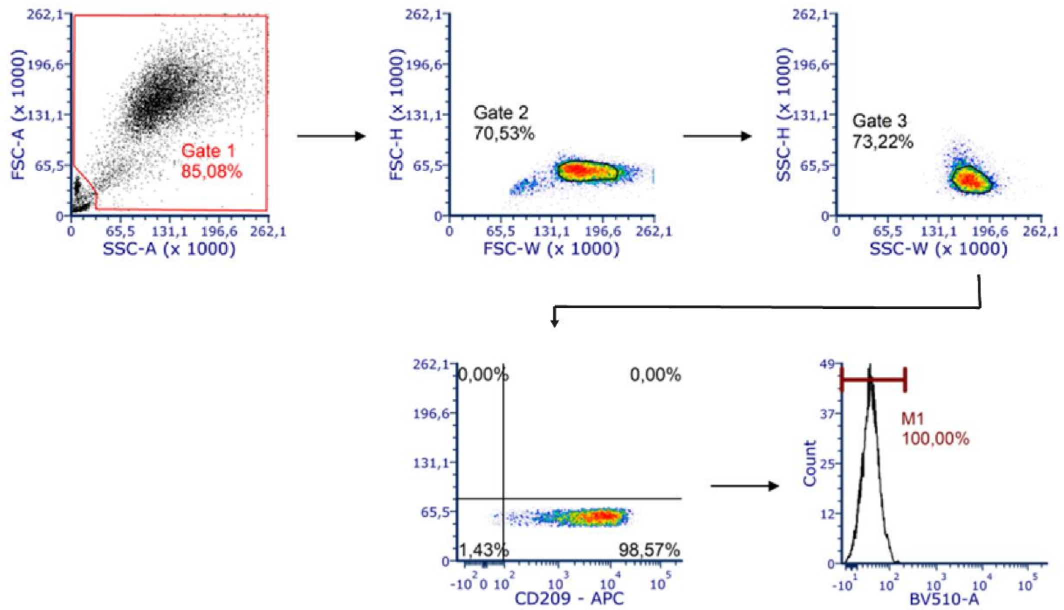

Supplementary Fig. S1. Gating strategy to identify human moDCs. Doublets were excluded by consecutive gating of FSC-H/FSC-W and SSC-H/SSC-W. MoDCs were selected based on the expression of the specific moDC marker DC-SIGN (CD209). Dead cells were excluded by using the Zombie Aqua Fixable Viability Kit.

Supplementary Table 1. Cytokine/Chemokine secretion in response to SDSE infections. Values are displayed in [pg/ml]. (unstim., unstimulated)

		Human serum			Fetal calf serum		
		unst.	S118	S118Δ*ska*	unst.	S118	S118Δ*ska*
IL-1β	donor 1	159.56	589.86	458.49	252.08	739.32	377.92
	donor 2	34.51	839.65	466.65	62.61	127.9	1058.15
	donor 3	39.74	94.03	59.27	5.4	532.14	496.04
	donor 4	2.69	208.64	123.46	2.69	468.55	182.78
	donor 5	2.69	16.95	14.1	2.69	15.67	46.29
	**Mean**	**47.838**	**349.826**	**224.394**	**65.094**	**376.716**	**432.236**
	SD	64.81	351.32	220.89	107.61	298.47	390.50
IFN-α2	donor 1	51.48	38.98	108.19	73.8	94.51	49.75
	donor 2	6.65	43.36	28.07	12.7	25.84	45.18
	donor 3	23.08	19.03	8.31	6.9	22.62	21.75
	donor 4	0.5	7.5	7.71	0.41	12.71	10.9
	donor 5	0.41	2.72	3.4	0.41	2.17	4.03
	**Mean**	**16.424**	**22.318**	**31.136**	**18.844**	**31.57**	**26.322**
	SD	21.67	18.27	44.12	31.15	36.38	20.37
IFN-γ	donor 1	55.32	79.66	191.98	112.8	54.16	55.78
	donor 2	14.3	153.28	74.81	12.65	27.79	83.57
	donor 3	85.63	24.19	15.04	4.99	51.65	31.2
	donor 4	3.75	15.65	29.22	2.04	13.83	11.39
	donor 5	2.04	8.65	18.09	2.04	2.8	3.75
	**Mean**	**32.208**	**56.286**	**65.828**	**26.904**	**30.046**	**37.138**
	SD	36.84	61.04	74.49	48.21	22.69	32.85
TNF	donor 1	1992.73	14161.88	11669.06	2565.77	52830.59	9584.98
	donor 2	493.38	79655.36	33954.97	927.81	34343.98	106759.68
	donor 3	340.35	23071.23	5474.34	109.13	105306.53	83119.28
	donor 4	66.24	87030.65	19545.57	66.24	120000	120000
	donor 5	66.24	24002.21	3593.02	66.24	37860.02	66056.99
	**Mean**	**591.788**	**45584.266**	**14847.392**	**747.038**	**70068.224**	**77104.186**
	SD	804.35	34780.13	12367.19	1081.01	39829.78	43117.76
MCP-1	donor 1	47995.91	12407.05	21866.01	38279.06	21873.21	18301.31
	donor 2	247.75	3032.61	906.18	2239.53	3559.29	5189.5
	donor 3	40315.13	86783.87	26645.07	27151.41	64116.8	88636.84
	donor 4	157.1	896.75	288.13	236.89	4639.75	2770.73
	donor 5	472.38	730.02	265.51	352.23	1720.27	2366.84
	**Mean**	**17837.654**	**20770.06**	**9994.18**	**13651.824**	**19181.864**	**23453.044**
	SD	24178.07	37212.00	13130.49	17859.31	26395.25	37018.28
IL-6	donor 1	4405.02	18493.92	31645.19	40319.87	37541.96	15439.05

	donor 2	736.2	30880.67	25747.06	6553.28	21225.01	50790.61
	donor 3	2459.64	54837	18943.11	2632.29	110000	110000
	donor 4	102.77	28126.82	14722.28	155.89	110000	79752.77
	donor 5	68.81	7228.61	971.93	80.76	12477.33	26875.28
	**Mean**	**1554.488**	**27913.404**	**18405.914**	**9948.418**	**58248.86**	**56571.542**
	SD	1866.09	17679.92	11693.72	17180.96	48090.89	38727.05
IL-8	donor 1	49486.27	55578.98	100477.45	107169.59	48442.57	13501.73
	donor 2	34693.6	73887.87	81552.67	86968.79	35253.41	48363.84
	donor 3	32765.72	120000	120000	7771.31	89874.14	120000
	donor 4	4940.86	31110.64	33002.94	7345.24	53651.37	21233.94
	donor 5	2578.86	10820.32	4188.72	2089.61	3902.17	10302.98
	**Mean**	**24893.062**	**58279.562**	**67844.356**	**42268.908**	**46224.732**	**42680.498**
	SD	20364.61	41984.68	48059.89	50582.36	31132.43	45744.58
IL-10	donor 1	84.11	248.72	1681.25	631.25	1536.59	115.67
	donor 2	27.17	578.12	236.4	48.84	183.42	987.64
	donor 3	199.56	991.06	148.03	54.62	1098.78	1256.22
	donor 4	32.01	221.54	126.17	22.54	1196.02	462.6
	donor 5	12.41	40.88	26.25	12.41	89.23	139.7
	**Mean**	**71.052**	**416.064**	**443.62**	**153.932**	**820.808**	**592.366**
	SD	76.77	375.33	695.88	267.41	646.50	511.35
IL-12p70	donor 1	15.81	802.25	262.91	17.44	4364.9	2905.45
	donor 2	5.15	4207.49	1523.78	6.62	10712.41	12156.53
	donor 3	59.78	389.15	73.19	3.08	7084.11	2745.5
	donor 4	1.32	1244.02	358.07	1.32	10668.78	6890.45
	donor 5	1.32	399.58	80.4	1.32	2005.05	4955.61
	**Mean**	**16.676**	**1408.498**	**459.67**	**5.956**	**6967.05**	**5930.708**
	SD	24.82	1603.58	607.16	6.77	3845.02	3870.74
IL-17A	donor 1	12.87	9.45	21.95	5.42	8.1	3.87
	donor 2	1.91	9.16	6.2	0.59	0.86	1.88
	donor 3	15.93	2.27	3.77	0.7	1.33	2.76
	donor 4	0.97	3.14	4.89	0.7	1.22	1
	donor 5	1.53	2.34	4.44	0.7	0.7	0.7
	**Mean**	**6.642**	**5.272**	**8.25**	**1.622**	**2.442**	**2.042**
	SD	7.17	3.70	7.71	2.12	3.17	1.30
IL-18	donor 1	999.08	1647.77	2340.31	1176.56	1262.2	877.55
	donor 2	418.37	2131.17	2591.07	503.18	591.22	1319.03
	donor 3	287.62	455.05	262.19	128.7	530.47	537.85
	donor 4	131.67	178.34	167.86	23.56	154.54	137.26
	donor 5	124.98	154.54	137.26	28.89	37.04	55.22
	**Mean**	**392.344**	**913.374**	**1099.738**	**372.178**	**515.094**	**585.382**
	SD	360.28	914.95	1250.93	490.63	480.30	526.25

IL-23	donor 1	191.67	5615.26	5401.28	917.76	18702.28	5368.71
	donor 2	95.84	13376.66	9938.48	138.24	4333.18	26617.29
	donor 3	19.21	1247.99	459.35	14.25	4382.75	9861.94
	donor 4	8.78	2897.21	886.85	8.78	13982.18	5571.91
	donor 5	8.78	834.65	100.66	8.78	1926.18	2308.12
	**Mean**	**64.856**	**4794.354**	**3357.324**	**217.562**	**8665.314**	**9945.594**
	SD	79.71	5152.30	4260.19	395.31	7272.09	9700.01
IL-33	donor 1	133.79	227.21	371.99	228.42	186.5	152.03
	donor 2	35.33	216.75	145.88	51.93	68.21	133.79
	donor 3	357	69.19	70.02	15.84	74.17	82.37
	donor 4	9.79	59.97	120.41	9.79	59.12	43.68
	donor 5	9.79	33.13	103.23	9.79	9.79	9.79
	**Mean**	**109.14**	**121.25**	**162.306**	**63.154**	**79.558**	**84.332**
	SD	147.67	92.98	120.42	94.04	64.96	59.67
